# Case report: Genetic defects in laminin α5 cause infantile steroid-resistant nephrotic syndrome

**DOI:** 10.3389/fped.2022.1054082

**Published:** 2023-01-11

**Authors:** Yoon Sunwoo, Naye Choi, Jeesu Min, Jihyun Kim, Yo Han Ahn, Hee Gyung Kang

**Affiliations:** ^1^ Department of Pediatrics, Seoul National University Children’s Hospital, Seoul, South Korea; ^2^ Department of Pediatrics, Seoul National University College of Medicine, Seoul, South Korea

**Keywords:** case report, LAMA5, laminin, SRNS, genetic nephrotic syndrome

## Abstract

Single gene pathogenic mutations have been implicated in up to 30% of pediatric steroid-resistant nephrotic syndrome (SRNS) cases, mostly in infantile patients. Among them is *LAMA5*, which has been recently discovered and encodes the laminin α5 chain. The laminin α5β2γ1 heterotrimer is an essential component of the glomerular basement membrane and is necessary for embryogenesis and immune modulation. Biallelic *LAMA5* variants have been identified in one adult and ten pediatric nephrotic syndromes (NS) patients with variable phenotypes. Biallelic truncating mutations in this gene have recently been proven to cause SRNS. Here, we present another case of infantile SRNS related to novel compound heterozygous variations of *LAMA5* (c.3434G > A, p.Cys1145Tyr and c.6883C > T, p.Gln2295*), the first reported case with one missense and one nonsense allele. A 10-month-old female patient presented with eyelid edema and massive proteinuria without any extrarenal symptoms or family history. The patient was diagnosed with SRNS. Renal biopsy revealed focal segmental glomerulosclerosis with widely effaced epithelial foot processes and a “moth-eaten” appearance. She progressed to end stage kidney disease (ESKD), requiring dialysis at 31 months of age, and underwent a deceased-donor kidney transplant at 6 years of age. Four months after transplantation, she developed Ebstein-Barr Virus (EBV) infection related to post-transplantation lymphoproliferative disorder (PTLD). After chemotherapy, the patient remained healthy with adequate renal function without disease recurrence for the past 7 years. We also identified previous cases of biallelic *LAMA5* variants associated with the nephrotic phenotype and analyzed the available clinical and genetic information. All reported patients had an onset of NS ranging from 3 months to 8 years, with no other syndromic features. Response to therapy and renal outcomes varied greatly; most patients exhibited steroid resistance, five progressed to ESKD, and two received kidney transplantation (KT). There was one report of PTLD. Our patient’s phenotype was markedly more severe than those with biallelic missense variants and somewhat less severe than those with two truncating variants. *LAMA5* defects may also play a role in PTLD, though no conclusions can be made with such limited cases. *LAMA5* should be considered a candidate gene for SRNS and should be actively tested in cases with no other genetic diagnosis.

## Introduction

Steroid-resistant nephrotic syndrome (SRNS) is a subtype of nephrotic syndrome (NS) characterized by proteinuria, hypoalbuminemia, and edema that does not respond to standard steroid therapy. Fifty percent of patients with SRNS are reported to develop end-stage kidney disease (ESKD) within 15 years, indicating a poor prognosis. Over 70 genes, often encoding proteins of the podocytes or the glomerular basement membrane (GBM), have been reported as causative factors of SRNS (1–3). Of those recently discovered, *LAMA5*, which encodes laminin α5, is the predominant alpha chain of laminin in the mature GBM (4).

**Figure 1:**
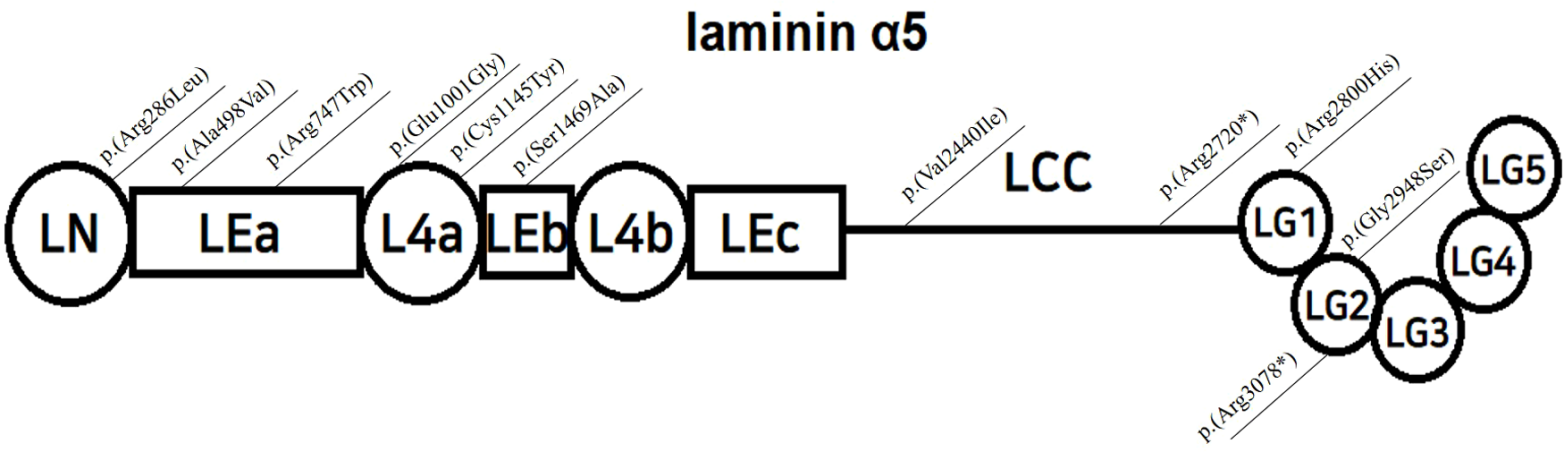
Structure of laminin α5, modified from Taniguchi Y, and localization of reported variants; LN=Laminin-N-terminal; mediates trimer polymerization LEa,b,c=Laminin-Epidermal Growth Factor-like domain a,b,c; L4a,b=laminin domain 4 a,b; LCC=long coiled-coil domain LG 1-5=laminin globular domain 1-5, interacts with integrins, dystroglycans, and syndecans.

Laminin-α5 is composed of three globular domains (LN, L4a, and L4b) and three rod-like elements (LEa, LEb, and LEc), followed by a long coiled-coil domain (LCC), ending with five laminin G -like domains (5) (LG 1–5, Figure 1). Together with laminin β2 and γ1, it forms the laminin α5β2γ1 heterotrimer by joining the LCCs. The LG domain of the heterotrimer interacts with integrins, dystroglycans, syndecans, and basal cell adhesion molecules, facilitating anchorage and mediating crosstalk between podocytes and GBM (4, 6, 7).

Defects in *LAMA5* have been associated with kidney disease in murine models since 2000, in addition to abnormalities in embryogenesis, extracellular matrix formation, and immune modulation (6, 8). *LAMA5* knockout mice are lethal during the embryonic phase, with disrupted glomerulogenesis (9). Mice with homozygous hypomorphic *LAMA5* alleles demonstrate hematuria, proteinuria, renal cysts, and progressive renal failure at 3–4 weeks of age (10). Podocyte-specific inactivation of *LAMA5* produced proteinuria in mice, their kidneys showing foot process effacement and a thick and thin “moth-eaten” appearance of GBM (11). Mice with biallelic *LAMA5* mutations in the L4a domain (Glu884Gly) showed reduced secretion of the laminin α5β2γ1 heterotrimer and, in turn, altered GBM composition (12). Following the first connection of *LAMA5* to human kidney disease in an adult female with focal segmental glomerulosclerosis (FSGS) in 2013 (13), ten additional cases of *LAMA5*-related recessive pediatric NS have been reported, with onset ages ranging from 3 months to 8 years, and various responses to treatment. Functional studies have revealed that biallelic truncating mutations of *LAMA5* are indeed the cause of NS (14).

Here, we report another rare case of infantile SRNS related to compound heterozygote variants of *LAMA5*, the only reported case with one missense and one nonsense allele.

## Case description

A 10-month-old female presented with eyelid edema and massive proteinuria and was admitted to an outside hospital in September 2007. She was born at a gestational age of 40 weeks and a birth weight of 2.94 kg, delivered vaginally without perinatal problems. The patient was the only child with no remarkable family history of renal disease. Upon admission, the patient was treated with a total dose of oral steroids for 4 weeks. Symptoms seemed to subside in the first 2 weeks, but edema and proteinuria worsened in the following 2 weeks. After 4 weeks of steroid treatment, the patient was transferred to our center for further management of infantile SRNS.

When transferred, her height was 69.5 cm (3–10 p), and her weight was 7.7 kg (10–25 p). She showed mild periorbital swelling and abdominal distension. The initial laboratory findings were as follows. Serum albumin 1.6 g/dl, serum BUN/creatinine 4/0.4 mg/dl, serum cholesterol 712 mg/dl, IgG/A/M respectively 236/33/131 mg/dl, and C3/C4 103/16 mg/dl. Serological testing was negative for HBV, HCV, HIV, and RPR/VDRL. Urine dipstick examination showed albuminuria (3+) and a random urine protein/creatinine ratio of 27.47 mg/mg. Kidney ultrasonography findings were unremarkable, with no signs of increased echogenicity or renal cysts. A kidney biopsy was performed, and its pathology was consistent with focal segmental glomerulosclerosis (FSGS) of the perihilar type. Four of 101 glomeruli (3.9%) showed global sclerosis, three (2.9%) showed segmental sclerosis, and five (5.0%) showed small crescentic lesions. Focal GBM abnormalities were noted ultra-structurally, with widely effaced epithelial foot processes and a moth-eaten appearance. The patient did not show any remarkable extrarenal abnormalities. Upon admission in November 2007, at the age of 12 months, she was tested for known mutations in the *NPHS2*, *WT1*, and *ACTN* genes using Sanger sequencing, but all were negative.

**Figure 2:**
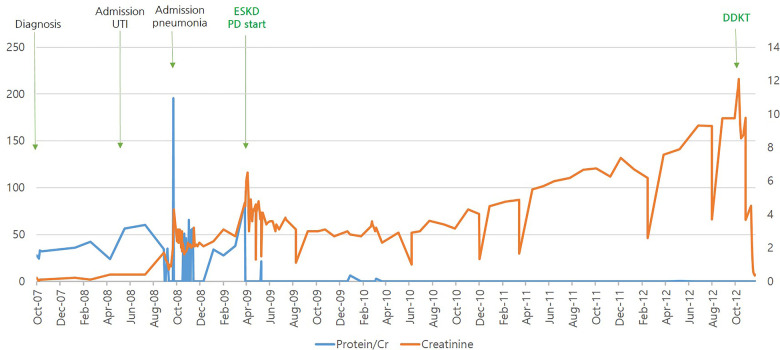
Patient's clinical course according to urine protein/creatinine and serum creatinine.

She was treated with oral steroids and angiotensin-converting enzyme (ACE) inhibitors, but her proteinuria and renal function worsened, along with occasional viral infections. The patient progressed to ESKD within 31 months and was started on peritoneal dialysis. She underwent a deceased-donor kidney transplant at 6 years of age. She then developed Ebstein-Barr Virus (EBV)-associated post-transplant lymphoproliferative disorder (PTLD) of the intestine 4 months after transplantation, with EBV titers rising to 166,571 copies/ml in the blood. The patient was seronegative for EBV before transplantation, and the kidney donor was seropositive for EBV. Ileo-colectomy was performed, followed by intensive chemotherapy for 3 years, and remission was achieved in 2015. At her most recent follow-up in July 2022, the patient had been off therapy for 7 years and had maintained adequate renal function without recurrence. The clinical course of the patient is shown in Figure 2.

In June 2021, new whole exome sequencing technology became available for use in still undiagnosed patients. Only then was the patient was found to have compound heterozygous variations of LAMA5 through whole exome sequencing. Both variants were predicted to be deleterious by *in silico* prediction; c.6883C > T (p.Gln2295*) is a nonsense variant classified as likely pathogenic (LP) by the American College of Medical Genetics, inherited from the father, and c.3434G > A (p.Cys1145Tyr) is a *de novo* missense variant classified as a variant of unknown significance (VUS).

## Discussion

This is the first case report of *LAMA5*-related nephropathy in Korea, the only reported case with one missense allele and one nonsense allele, and the second case of PTLD. This case fits well with previous reports, with the onset of SRNS at 10 months and progression to ESKD at 31 months with no other syndromic features. Table 1 summarizes the characteristics of patients with *LAMA5* variants for whom clinical information was available. Reported pediatric patients with *LAMA5* mutations had an onset of NS ranging from 3 months to 8 years. Except for the two Japanese siblings with bilateral cataracts and one syndromic case with Arg286Leu variation, all patients exhibited no extra-renal manifestations or syndromic features. Most patients were steroid-resistant and progressed to ESKD by ages 1–6. Renal histology revealed by renal biopsy was consistent with previous reports of both mouse models and clinical data: FSGS, foot process effacement, and thick and thin “moth-eaten” GBM.

**Table 1:** Characteristics of reported patients with LAMA5 mutation and nephrotic phenotype

ID	Sex	Onset	ESKD	KT	Variant	Domain		Biopsy	SR	Extrarenal	Race	Comments
1(1)	M	3mo	1.6yr	7yr	p.(Arg3078*)	LG2	P	-	+	O cataract	Japanese	Rejection 3yr after KT eGFR 29 ml/min/ 1.73m2 at 16yr
2(1)	F	4mo	3.5yr	X	p.(Arg3078*)	LG2	P	-	+	O cataract	Japanese	Sibling of Pt. 2 on CAPD at 8yr
3(1)	F	6mo	3.3yr	4yr	p.(Arg2720*)	LCC	P	DMS	+	X	Japanese	Normal eGFR at 8yr
**4**	**F**	**10mo**	**3.1yr**	**6yr**	**p.(Cys1145Tyr)** p.(Gln2295*)	**L4a** **LCC**	**VUS** **LP**	**FSGS**	**+**	**X**	**Korean**	**EBV-PTLD 4mo after KT** **Normal eGFR at 15yr**
5(2)	F	1.5yr	6yr	X	p.(Glu1001Gly)	L4a	VUS	FSGS	+	X	Egyptian	Sibling of Pt. 8 Death before KT
6(2)	M	22mo	-		p.(Arg747Trp)	LEa	VUS	-	-	X	Turkish	Sibling of Pt. 6 4 relapses, Normal eGFR at 9yr
7(3)	M	2yr	2yr		p.(Arg286Leu)	LN	P	FSGS	+	**O**	Italian	EBV-PTLD 3 yrs after KT cystic dysplastic RK, dysplastic LK, bilat VUR GI, skeletal anomalies, HL
8(2)	M	3yr	-		p.(Arg747Trp)	LEa	VUS	-	-	X	Turkish	3 relapses, Normal eGFR at 11yr
9(2)	M	4yr	-		p.(Gly2948Ser)	LG2	VUS	-	+	X	Arabic	Frequent relapses
10(2)	M	4yr	-		p.(Glu1001Gly)	L4a	VUS	FSGS	+	X	Egyptian	
11(1)	F	8yr	-		p.(Ala498Val) p.(Arg2800His)	LEa LG1	LP LP	FSGS	+	X	Japanese	Normal eGFR at 9yr
12(4)	F	27yr	-		p.(Ser1469Ala) p.(Val2440Ile)	LEb LCC	VUS B	FSGS	?	X	African American	

Modified from Taniguchi et al. VUS, variant of uncertain significance; LP, likely pathogenic; P, pathogenic; FSGS, focal segmental glomerulosclerosis; DMS, diffuse mesangial sclerosis; SR, steroid resistance; eGFR, estimated glomerular filtration rate; RK, right kidney; LK, left kidney; VUR, vesicoureteral reflux; HL, hearing loss; GI, gastrointestinal; PD, peritoneal dialysis.

The variants c.6883C > T (p.Gln2295*) and missense variant c.3434G > A (p.Cys1145Tyr) have not been previously reported. This missense variant is in a highly evolutionarily conserved sequence in zebrafish. The variants were not found in control databases such as ClinVar, gnomAD, ExAC, and 1000G (15). Twelve out of 19 tools in Varsome predicted the truncating variant to be deleterious, and five of the eight indicated the *de novo* variant to be harmful (16). Staining for laminin α5 expression would have been a reasonable method to prove pathogenicity but could not be performed because the biopsy sample was outdated. While no functional study was conducted to verify these variants’ pathogenicity, we assume that this is another case of LAMA5-related NS considering striking phenotypic compatibility. However, the significance of one missense variant remains uncertain.

While our patient had only one truncating allele, her phenotype was more severe than those with biallelic missense variants and somewhat less severe than those with two truncating variants. This missense mutation is in the L4a subunit, where other pathogenic missense variants have been reported in humans and mice. Analysis of a murine model generated by Falcone et al. with an L4a domain variant (Gly3685Arg) showed depletion of laminin α5 and different protein composition and organization of the GBM compared to wild mice (12). Falcone et al. postulated that altered GBM properties might form a more fluid matrix that subjects podocytes to more mechanical stress, leading to the nephrotic phenotype. The specific pathogenic mechanism and role of this protein domain have yet to be elucidated. The truncating mutation is located in the LCC domain, which is essential for laminin trimer formation, as described by Taniguchi et al. (14). Recombinant mice with truncating variants in this domain secrete truncated laminin α5, which can trimerize, form mutant laminin α5β2γ1 trimers, and avoid lethality. However, these mice showed insufficient levels of laminin α5 in the GBM and exhibited heavy proteinuria, concordance with the patient phenotype of infantile NS (17).

According to the International Pediatric Nephrology Association SRNS guidelines, comprehensive genetic panel testing should be performed in all children with primary SRNS if available (18). After initial steroid treatment, it is reasonable to add renin-angiotensin system (RAS) inhibitors until the diagnosis is confirmed. Once monogenic SRNS is proven, immunosuppressive treatments, such as steroids or calcineurin inhibitors, should be discontinued as they are mostly ineffective, in contrast to non-genetic SRNS. RAS inhibition should be continued to decelerate progression to ESKD and reduce proteinuria. There seems to be a wide variation in nephrotic phenotypes related to *LAMA5*, with some steroid-sensitive forms. Still, among the steroid-resistant, the few cases reported to use immunosuppressants showed no response, especially those with infantile-onset (14, 19). Our patient received no further immunosuppressive treatment other than initial steroids and ACE inhibitors under solid suspicion of genetic SRNS, which was the reasonable approach.

*LAMA5* is implicated in transplant tolerance and immunity by regulation of T cells in mouse studies (8). The post-kidney transplant (KT) course of *LAMA5*-related NS has been described in four cases, including this report. As expected from genetic NS, none recurred after KT (20); however, EBV-related PTLD occurred in two, accounting for half of the transplant cases. The Arg286Leu case was diagnosed at age 5 and 3 years after KT (19); in our patient, it was diagnosed only 4 months after KT. Considering that the known incidence of PTLD is 1%–4% in pediatric KT recipients (21, 22), the relatively high incidence in these patients is intriguing. However, regarding our patient’s pre-transplant seronegative status for EBV and small number of total cases it is premature to assume any correlation.

In summary, we report a Korean case of infantile SRNS associated with *LAMA5* variants and the first published case with one missense and one nonsense variant. Because identifying a precise, monogenic diagnosis helps guide therapy and predict recurrence risk after renal transplantation, *LAMA5* should be recognized as a candidate gene and actively tested in cases of nephrotic patients with no other genetic diagnosis.

## Data Availability

The original contributions presented in the study are included in the article/Supplementary Material, further inquiries can be directed to the corresponding author.
